# Social difficulties and care burden of adult Duchenne muscular dystrophy in Japan: a questionnaire survey based on the Japanese Registry of Muscular Dystrophy (Remudy)

**DOI:** 10.1186/s13023-024-03087-z

**Published:** 2024-04-30

**Authors:** Madoka Mori-Yoshimura, Keiko Ishigaki, Yuko Shimizu-Motohashi, Naoko Ishihara, Atushi Unuma, Sumiko Yoshida, Harumasa Nakamura

**Affiliations:** 1 Department of Neurology, National Center Hospital, National Center of Neurology and Psychiatry, 4-1-1 Ogawahigashi, Kodaira, Tokyo, 187-8551 Japan; 2 Department of Pediatrics, Tokyo Women’s Medical College, Tokyo, Japan; 3 Department of Child Neurology, National Center of Neurology and Psychiatry, National Center Hospital, Tokyo, Japan; 4 Medical Genome Center, National Center of Neurology and Psychiatry, 4-1-1 Ogawahigashi, Kodaira, Tokyo, 187-8551 Japan; 5 Department of Laboratory Medicine, National Center Hospital, National Center of Neurology and Psychiatry, 4-1-1 Ogawahigashi, Kodaira, Tokyo, 187-8551 Japan; 6 Department of Psychiatry, National Center Hospital, National Center of Neurology and Psychiatry, 4-1-1 Ogawahigashi, Kodaira, Tokyo, 187-8551 Japan; 7 Department of Clinical Research Support, National Center Hospital, National Center of Neurology and Psychiatry, 4-1-1 Ogawahigashi, Kodaira, Tokyo, 187-8551 Japan

**Keywords:** Adult Duchenne muscular dystrophy, Dystrophinopathy, Patient registry, Questionnaire, Education, Bullying, Employment, Family care, Household income

## Abstract

**Background:**

Little is known about the social difficulties and health care needs of adult Duchenne muscular dystrophy (DMD) patients in Japan, as well as the financial and physical stress experienced by their caregivers. This study aimed to clarify the social circumstances surrounding adult DMD patients and assess the degree of involvement of family members in their care and the associated economic burden of the disorder in Japan.

**Methods:**

Adult DMD patients were identified through the Registry of Muscular Dystrophy (Remudy) in Japan and invited to complete a questionnaire together with a caregiver. Data on health care use, quality of life, work status, informal care, and household expenses were collected to estimate the costs associated with DMD from social and caregiver household perspectives.

**Results:**

In total, 234 (63.7%) of 367 adult DMD patients (mean age, 27.4 ± 6.0; range, 20–48 years) completed the questionnaire. Of these, 38 (21%) had developmental disorders (mental retardation, autism, and learning disorders), 57 (33%) experienced bullying in school, and 44 (77%) indicated the reason for bullying to be their physical handicap. Employment histories were noted by 72 (31%), although 23 (10%) lost their jobs mainly due to physical difficulties. Of the 234 patients, 164 (74%) lived with their relatives, and 78% of care time was supplied by family members, in particular, their mothers. The mean rate of care work provided by family members was 81%. Household income of families with an adult DMD patient was lower, whereas the rate of living with parent(s) and grandparent(s) was higher, in comparison with the general Japanese population.

**Conclusions:**

Adult DMD patients in Japan experience many social difficulties from childhood up to adulthood. As adults, many DMD patients experience bullying and workplace difficulties. Families were found to provide most of the care and financial support for DMD patients. Our results suggest the need to improve public patient care systems, including financial support, to address the physical and economic burdens of care for adult DMD patients in Japan.

**Supplementary Information:**

The online version contains supplementary material available at 10.1186/s13023-024-03087-z.

## Background

Duchenne muscular dystrophy (DMD, OMIM 310200) is an incurable, X-linked recessive form of muscular dystrophy caused by mutations in the dystrophin gene (*DMD*) located on chromosome Xp21.2 [1, 2]. The prevalence of DMD is less than 10 cases per 100,000 males and is consistent across countries [3]. The earliest symptoms of DMD are difficulties with climbing stairs, a waddling gait, and frequent falls; patients present with these symptoms at around 2–3 years of age [3]. Most patients become wheelchair dependent at around 10–12 years of age and need assisted ventilation at around 20 years of age [3]. Cardiac and respiratory complications are observed, especially with disease progression, and if left untreated, DMD typically leads to death in the late teens [4, 5].

With optimal care, death occurs in most patients with DMD between 20 and 40 years of age due to cardiac and/or respiratory failure [1]. With intervention, including medical management (e.g., respiratory and cardiac care), some patients can live into their 30 s and 40 s. The prognosis of DMD has improved through targeted interventions such as respiratory support, physical and respiratory rehabilitation, and cardiac management, which address known disease complications. Recent advances in multidisciplinary care, involving neurologists, rehabilitation specialists, neurogeneticists, pediatricians, home-care nurses, and primary-care physicians, have also extended the lives of DMD patients. For example, the mean age at death increased from 18.9 ± 4.1 years to 31.1 ± 5.4 years after the introduction of mechanical ventilation and cardiac medication, according to a single center survey conducted in Japan [6]. According to the Muscular Dystrophy Clinical Trial Network in Japan, the proportion of adults among the entire DMD population was 52.4% (856 out of 1633) in key hospitals of muscular dystrophy (MDCTN, unpublished data), suggesting that more adult DMD patients are now surviving longer.

Recent therapeutic approaches that aim to restore the missing dystrophin protein or address secondary pathology have received regulatory approval, and many other approaches are in clinical development. Gene-addition, exon-skipping, stop codon readthrough, and genome-editing therapies can restore the expression of a partially functional dystrophin protein, whereas other therapeutic approaches aim to improve muscle function and quality by targeting pathways involved in DMD pathogenesis [6]. To date, adeno-associated virus-mediated gene therapy (Delandistrogene moxeparvovec), exon skipping therapy (Eteplirsen, Golodirsen, Casimersen, Viltolarsen), and stop codon readthrough (Ataluren) have been approved in the United States and/or European Union [7]. Improvement of life expectancy is anticipated with further advances in the development of therapeutics.

There are also many unsolved problems which need to be addressed. For instance, central nervous system involvement of the disease is an important but underestimated issue. Brain comorbidities can have a greater impact on the family than limited mobility for people living with DMD [8], although there have been no clinical trials or agents which target the brain of DMD patients. The economic burden and care burden of patients and families have also been reported in many countries [9–11]. Infrequent employment among adult DMD patients [12] contributes at least in part to the economic burden. These unsolved problems may exacerbate as the duration to receive care becomes longer due to an increase in life expectancy.

Nonetheless, little research has been conducted on the life circumstances, care provision, and economic burden of DMD patients, their families, and caregivers in Japan. Two studies on this topic have reported on the heavy care burden of families, especially mothers, and suggested practical support for parental caregivers who experience a marked increase in the duration of their caregiving role while facing their own aging-related challenges [13, 14]. To our knowledge, no study has reported on the school life, working, and financial aspects of DMD in Japan.

To clarify potential relationships of DMD with social difficulties experienced by DMD patients, the educational environment, the workplace, care, and economic aspects of DMD, we conducted a questionnaire survey of DMD patients registered with Remudy, a patient registry of muscular dystrophy in Japan.

## Results

### Participant characteristics at study entry (Fig. 1, Table 1)

**Fig. 1:**
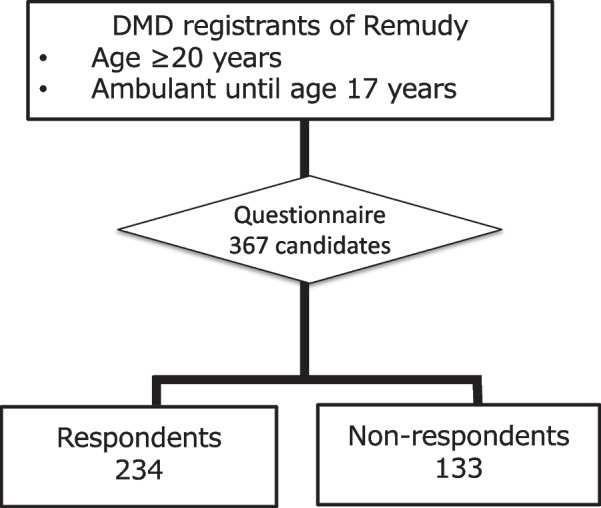
Patient recruitment. We sent questionnaire forms to 367 registrants with dystrophinopathy who were aged ≥ 20 years and became non-ambulant by age 12, or age 14 if they had been treated with steroids. Of these, 234 patients responded

**Table 1:** Participant characteristics, ambulation, and ventilator use

		n	(%)	Age, mean ± SD (median, range)
Respondent		234	63.7	27.4 ± 6.0 (25, 20–48)
Respondent	Patient	64	27.1	
	Representative*	172	72.9	
Age at loss of ambulation		228	97.4	9.9 ± 1.7 (10, 5–14)
Age when starting wheelchair use		229	97.9	9.5 ± 1.6 (10, 5–14)
Ventilator use		186	79.5	
Age when starting ventilator use		191	81.6	18.8 ± 3.7 (18.5, 10–34)
	Part-time NPPV	95	40.6	
	Full-time NPPV	71	30.3	
	TPPV	25	10.7	
	No answer	9	3.8	

^*^Representatives: mother, 129; father, 35; caregiver, 3; aunt, 2; parents, 1; grandfather, 1; unknown, 1

*NPPV* Non-invasive positive pressure ventilation, *TPPV* Tracheostomy positive pressure ventilation

At the end of September 2022, a total of 367 patients with DMD who fulfilled the inclusion criteria had been registered with Remudy. Among them, 234 (63.7%) participated in the present study. Respondents comprised 62 patients (26.5%) and 172 representatives (73.9%). The breakdown of representatives was 129 mothers, 35 fathers, three caregivers, two aunts, one parent pair (mother and father), one grandfather, and one unknown. Mean age of participants was 27.4 ± 6.0 years (range, 20–48; median, 25).

A total of 191 (81.6%) participants used ventilators. The mean age at loss of ambulation was 9.9 ± 1.7 years (range, 5–14; median, 10), and that for the first use of a wheelchair was 9.5 ± 1.6 years (range, 5–14; median, 10). There were 95 (40.6%) part-time noninvasive positive pressure ventilation (NPPV) users, 71 (30.3%) full-time NPPV users, and 25 (10.7%) tracheostomy positive pressure ventilation (TPPV) users.

### Developmental disorders, problematic behavior, school life, and bullying (Table 2, 3, Additional file 1: Table 1)

**Table 2:** Bullying victimization during school and its influence on present mental status

			n	%
Bullying experience	Yes		74	31.6
	No		139	59.4
	No answer		21	9.0
	Perceived reason for being bullied*	Muscular dystrophy (physical reasons)	56	75.7
		Communication problems	16	21.6
		Learning problems	4	5.4
		Intellectual/developmental problems	3	4.1
		Others/no answer	15	20.3
Timing of bullying*	Nursery/Kindergarten	10	13.3	
	Elementary school		66	88.0
	Regular class		42	
	School for disabled children		13	
	Other/no answer		11	
	Junior high school		15	20.0
	Regular class		7	
	School for disabled children		6	
	Other/no answer		2	
	High school	1	1.3	
	No answer	1	1.3	
Influence of bullying on present mental status	Yes		29	
	Type of influence	Negative	13	
		Positive	7	
		No answer	9	
	No		46	

*Multiple answers accepted

**Table 3:** School type, academic performance, and assistant in school

			Elementary school		Junior high school		High school	
			n	%	n	%	n	%
School for disabled children			82	34.6	146	62.5	174	74.3
	Due to physical disability		72	30.8	120	51.3	169	72.2
	Due to intellectual disability		5	2.1	2	0.9	5	2.1
	Due to both physical and intellectual disabilities		5	1.7	24	10.3	0	0
Regular school			133	56.8	77	34.4	47	20.1
	Academic performance	Good	41	30.8	22	28.6		
		Fair	71	53.3	43	55.8		
		Poor	16	12.0	13	16.9		
		Others/no answer	5	3.8	1	1.3		
Others/no answer			20	8.5	11	4.7	13	5.6
Regular assistant present in school	Yes		123	52.6	90	38.5		
		Dispatched from Board of Education	92	74.8	71	30.3		
		Mother	20	16.3	7	3.0		
		Care worker	2	1.6	2	0.9		
		Other	12	9.8	13	5.6		
	No		49	39.8	60	25.6		
	No answer		62	26.5	84	35.9		

Forty-two (17.9%) participants were diagnosed with developmental disorders. These developmental disorders included intellectual disabilities (26; 11.1%), autism spectrum disorders (16; 4.8%), learning disorders (four; 1.7%), and others (five; 2.1%). Six participants with intellectual disabilities also had autism spectrum disorders.

Thirty-four participants experienced problematic behavior such as domestic violence (n = 15), cutting class (n = 5), suicide attempts and/or self-mutilation (n = 1), and violent incidents (n = 1).

With regard to academic background, four participants (1.6%) completed junior high school, 156 (66.6%) completed high school, 32 (13.7%) completed university/college, and eight (3.4%) completed graduate school. Nineteen participants (8.1%) were current university or college students.

Eighty-two (34.6%), 146 (62.5%), and 175 (74.3%) participants had attended elementary school, junior high school, and high school for disabled children, respectively. In total, 186 participants had attended schools for disabled children/students, whereas 36 participants had never attended such schools. Reasons for attending special schools were physical disability (elementary, junior high, and high school: n = 72, 120, and 169, respectively), intellectual disability (n = 5, 2, and 5, respectively), or both (n = 5, 24, and 0, respectively). Of participants who had attended regular school classes, 16 (12.0%) and 13 (16.9%) had unsatisfactory academic performance in elementary school and junior high school, respectively (Table 2).

A total of 148 (63.2%) participants were granted the requests they made to schools for various accommodations, as follows: installation of or permission to use elevator (n = 145), renovations (n = 79, including installation of, e.g., slope, handrail, toilet), consideration of or assistance with mobility transfers (n = 121, including arrangement of classroom or assistance when moving within school). Ninety-two (39.0%) participants at elementary schools and 70 (30.5%) at junior high schools required an assistant. Among assistants, 20 (8.5%) at elementary schools and seven (3.0%) at junior high schools were mothers. (Additional file 1: Table 1).

Up until graduation, 74 (31.6%) participants experienced bullying, of which 56 indicated that their physical handicap due to muscular dystrophy was the reason for being bullied. During the obligatory education period, 13 of 66 participants in elementary school for disabled children, and six of 15 participants in junior high school for disabled children, were bullied (Table 3).

#### Employment (Table 4)

**Table 4:** Current and past work experience and reasons for not working/retiring early

	n (%)	Reason for not working		n (%)
With work experience	72 (30.8)			
Currently working	Yes	49 (20.9)		
	No	23 (9.8)	Physical	17
			Other	6
Without work experience	151 (64.5)	Physical disability		104 (44.4)
		Training/student		21 (8.9)
		Intellectual disability		11 (4.7)
		Personal reasons		6 (4.0)
		Others, no answer		9 (3.8)
No answer	11 (4.7)			

A total of 72 (30.8%) participants had work experience, 151 (64.5%) had never worked, and 49 (20.9%) were currently working. The most frequent reasons for having never worked included physical handicap (n = 104). Three participants gave up to work because a public care worker could not assist them during working hours. Among participants who had work experience, 23 no longer worked. Physical reasons were most frequently cited for not working (n = 17/23). Other reasons included preparing for the next job (n = 4) and personal reasons (n = 2).

### Daily care, physical burden, and financial burden (Table 5, Fig. 2)

**Table 5:** Cohabitants, caregivers, care provided by family members, and estimated loss of income due to care

				n	%
Cohabitant	Yes	Relatives		164	70.1
			Parents with/without siblings	96	41.0
			Parents and grandparent(s)	28	12.0
			Mother with/without siblings	18	7.7
			Father with/without siblings	2	0.9
			Mother and grandparent(s)	4	1.7
			Father and grandparent(s)	1	0.4
			Siblings	3	1.3
			Other relatives	2	0.9
		Spouse		1	0.4
		No answer		9	3.8
	No			23	9.8
			Lived alone	4	1.7
			Hospitalized/residential care	16	6.8
			Other	3	1.3
	No answer			49	20.9
Primary caregiver*	Mother			139	59.4
	Father			47	20.1
	Other relatives			7	3.0
Care provided by family members (%, hours)			78.8 ± 21.2 (5–100, median 90, n = 117)		
Unpaid care time by family members (hours)			15.6 ± 7.6 (0.8–24, median 16, n = 133)		
Care time ratio among family members (%)*	Mother			124	74.6
	Father			99	30.8
	Grandparent(s)			19	19.5
	Siblings			23	10.1
	Other			3	26.7
Estimated loss of income (yen/year)			2,614,443 ± 2,3205,29 (0–12,000,000, median 2,000,000, n = 76)		

*Multiple answers accepted

**Fig. 2:**
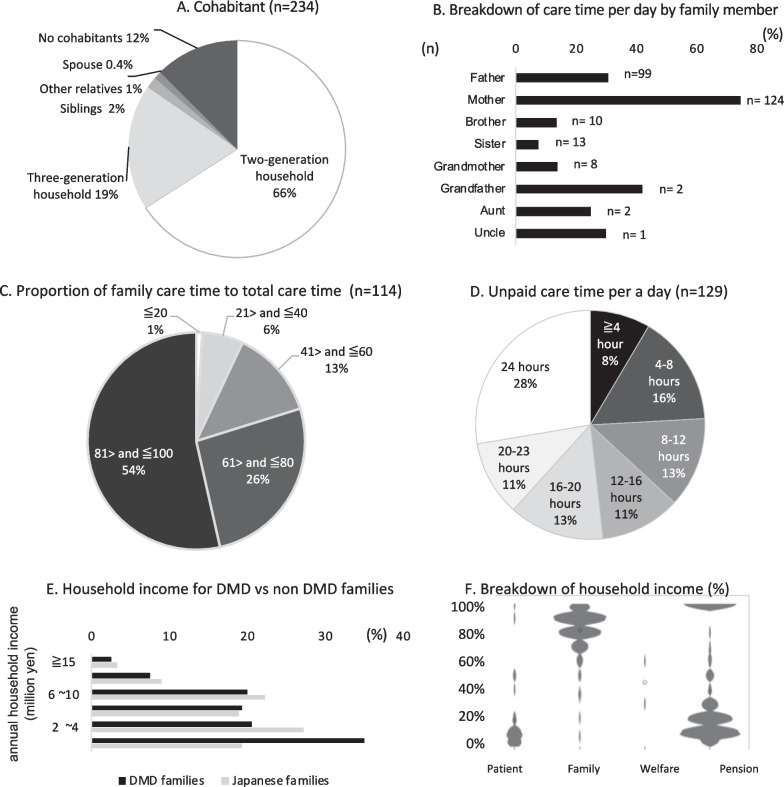
Care and household status. **A** Cohabitants of DMD patients. The chart shows the proportion (%) of each category based on the answers provided by cohabitants; 66% of patients lived in two-generation households and 19% lived in three-generation households. Only one participant lived with a spouse. **B** Breakdown of care time provided by family. Parents, especially mothers, supplied most of the care time. **C** Proportion of family care time to total care time. The most frequent answer was > 80%. **D** Unpaid care time per day; 28% answered “24 h”. **E** Household income for DMD vs non DMD families. Annual household income of DMD families (black column) and the general Japanese population (gray column). DMD families had lower incomes than the general Japanese population. **F** Income ratio of DMD family members. Most of the income was from parent (s); participants contributed to a portion of the household income with their disability pensions

A total of 164 patients lived with their relatives; four lived alone, and 16 lived in hospitals or received residential care (Table 5). Most patients (66%) lived in two-generation households (i.e., patient and parents (s) with or without siblings), and 19% lived in three-generation households. In other words, 80% of all patients lived with their parents (Fig. 2A).

The main caregiver was most often parent(s), in particular, mothers (n = 139). Among care time provided by family members, 74.6% was provided by mothers and 30.8% by fathers (Fig. 2B). Most of the care time was provided by the family (n = 117; mean, 78.8 ± 21.2%; range, 5–100%; median, 90%, Fig. 2C), and unpaid care time provided by the family was 15.6 ± 7.6 h per day (range, 0.8–24; median, 16 h; Fig. 2D).

The median household income range was 2,000,000–4,000,000 yen (corresponding to about US$19,000–37,000 or 16,000–32,000 euro based on conversion rates of US$1 = 107.19 yen and 1 euro = 126.04 yen on the day of questionnaire dissemination), which was lower than the mean household income in Japan of 5,458,000 yen/year (corresponding to about US$51,000 or 43,000 euro) [15]. Higher household incomes were less frequent and household incomes of ≤ 2,000,000 yen were more frequent among DMD families. Twenty-seven patients (11.4%) contributed to the household income, with an average of 18.6% of household incomes of these families being provided by the patients’ salary and 81.0% being provided by other family members (Fig. 2F).

## Discussion

In this study, we conducted a nationwide survey of adult DMD patients and obtained several new insights regarding adult DMD life and care in Japan, among which is the urgent need to overhaul the current care system for DMD patients. In Japan, family care at present is mainly provided by relatively young parents, mothers as housewives, cohabiting grandparents, and siblings, all of whom serve as caregivers throughout the patient's life, and on a single income provided by fathers. Thus, the care and costs related to DMD patients are being supported to a great extent by families throughout their school years until adulthood. Notably, although traditional three-generation families accounted for only 5.9% of all Japanese families in 2016 [16], 20.8% of DMD families included grandparent(s) as cohabitants, with grandparent(s) providing about 19.5% of the care time. This suggests that the traditional three-generation family structure may be advantageous for DMD care at present, with grandparents contributing to care for their grandchildren.

The need for an urgent overhaul of the care system is underscored by rapid changes occurring in Japanese society, in which both patients and families are aging, and the number of siblings in families are decreasing. In addition to the lengthening of lifespan of DMD patients, the age at which Japanese women give birth is increasing. In 1985, 66% of mothers were under age 30 when giving birth to a first child, but by 2022, this percentage dropped to 36%, with a reduction in number of births between the two years from 1,431,577 to 770,747 [17]. This translates to older mothers and fewer siblings, resulting in a situation in which parents need to care for patients longer, up to the point at which their age makes it no longer feasible to provide care.

The imbalance in care provided by mothers and fathers might be attributed to gender inequity, including wage disparities in Japan, with men generally earning a significantly higher wage than women, and the traditional mindset that providing care is a woman’s responsibility. It is thus common in Japan for mothers to stop working or not work at all in order to care for their children. The lower income of DMD families may be partly explained by the fact that most of the care and school assistance for DMD patients is provided by parents, in particular, mothers, rather than by non-family caregivers. However, given that 70.0% of married couples (with wives aged ≤ 64 years) are double income families in Japan [18], the single-income model (with income provided only by fathers) is also changing.

To solve the future shortage of care by family members, promoting public resources and increasing labor and funds for the care system may be required. Moreover, the traditional values of Japan make it difficult to ask for help from others and to use public resources to resolve family matters, such as the care of and financial support for disabled family members. Many people, including medical specialists of muscular dystrophy, feel hesitant to request public care for DMD, given the limited amount of human and financial resources available for care systems at present. It also will be important to promote inclusive education and educate the public on the benefits of mutual help in society with the aim of improving the understanding of patient care and social welfare. Efforts should also be made to change the traditional mindset that family members should provide all care without relying on social welfare systems.

Although it is difficult to directly compare our results with those of other countries due to differences across countries, such as cohabitation trends, culture, and available social support and welfare systems, it is informative to gain an understanding of the overall DMD care situation. According to a study of adult DMD patients in European countries, the rate of living independently or with families differed across countries, with all DMD patients living with family in Eastern Europe and 60.5% of patients living independently in Denmark [12]. The present study found that only four (1.7%) participants lived alone, and only one (0.4%) lived with a spouse. This may reflect the differences in care systems and welfare adequacy across countries. The lower independence of DMD patients in the present study, similar to that of Eastern European countries, might be attributed to differences in family members living with the patients. For instance, 35.8% of Japanese adults with alive parent(s) live with their parent(s) [16], which is a higher rate than that reported for other European countries [19]. For countries experiencing forthcoming social changes, such as changes in the composition of the population or an increase in gender equality as in Japan, the findings of the present study and insight gained on potential solutions may be informative.

We showed that some DMD patients earned their own salary and contributed to the household income in Japan. Specifically, 30.8% of DMD patients had work experience and covered their own expenses; 20.9% were working at the time of the questionnaire survey, which is a higher proportion than that reported in Canada [19] and European countries [12]. On the other hand, most patients were temporarily dependent on their family’s income. These findings suggest that more patients are now living long enough to earn a salary, and that developing support and informational tools for such patients may further enable their entry into the workforce. This may partially improve the relatively impoverished situation of DMD families.

Another observation of the present study was that DMD patients are at risk of being bullied and exhibit problematic disorders. These issues are also experienced by patients with Becker muscular dystrophy (BMD) [20, 21], a similar yet milder progressive muscular dystrophy compared to DMD [22]. The main reason of being bullied for both DMD and BMD patients was physical and/or developmental and intellectual problems, possibly due to the central nervous system involvement of dystrophinopathy. A surprisingly large number of DMD patients had experienced bullying (31.8%), especially considering that teachers were expected to provide an appropriate school environment and school assistants were present in most cases. In other words, bullying had occurred even while under the care of teachers and assistants. Among the general population in Japan, bullying reportedly is experienced by 47.7 per 1000 (4.8%) disabled children/students from elementary school to high school [23], indicating that the ratio of bullying reported by DMD patients in this study is high. We also found that bullying of DMD children/students was reported even in schools for disabled children during the obligatory education period. This may suggest the need for more attentive custody by parents and teachers, as well as the need to educate other students about inclusive education and diversity. In order to reduce the incidence of bullying experienced by DMD patients, the launching of governmental programs aimed at addressing the inappropriate (bullying) behavior of other students may be needed. Notably, the proportion of bullied DMD patients, however, was lower than that of BMD patients registered with Remudy (45.6%) [20]. This might be explained by the greater severity of DMD, as well as the earlier diagnosis and coming out of the disease.

The present survey also revealed that some DMD patients exhibit problematic behavior, similar to BMD patients [20, 21], suggesting again that DMD and BMD patients share similar issues. Problematic disorders are often related to developmental and intellectual disorders, and central nervous system disorders of dystrophinopathy together with social difficulties in school can be another possible source. Given the finding that problematic behavior in BMD patients was correlated with psychiatric disease [20], doctors of DMD patients with problematic behavior should carefully monitor their patients for psychiatric disease.

This study has several limitations. First, the questionnaire was subjective and elicited self-reported information. Thus, data used in the present study may not have been as objective as those used in other studies. Second, the recruitment of participants through a patient registry is a potential source of selection bias, as this group may be particularly motivated to get involved and more severely affected by the disorder. In addition, not all participants answered all questions, and the answer rate was sometimes lower than expected. Third, it is difficult to fully scrutinize psychiatric and developmental problems through a questionnaire survey, as previously reported [21]. Direct interview studies with DMD patients are warranted as a complement to the present study.

In conclusion, family members provide most of the care for DMD patients, as well as financial support. Our findings suggest the need for more discussions on welfare adequacy and employment promotion for persons with disabilities in Japan. In addition, adult DMD patients in Japan had experienced bullying and workplace difficulties, underscoring the urgent need to alert parents and teachers of this situation.

## Methods

### Objective

This study aimed to clarify the social circumstances surrounding adult DMD patients in Japan by assessing patient background, education and school life, employment, and household structure, and to estimate the involvement of family in patient care and the economic burden of the disorder.

### Patient registry, organization, registration method, data collection, and ethics approval

Information relating to the development and management of Remudy, the registration method, and data collection was published previously [24]. The flow of patient recruitment is shown in Fig. 1. Approval for the study was obtained from the Medical Ethics Committee of the NCNP (A2021-057). Study objectives, design, risks, and benefits of participation were explained to all participants, and their written informed consent was obtained prior to enrollment.

### Participant characteristics

Participants were registered patients with DMD who were aged ≥ 20 years. Of these, patients who were non-ambulant at age 12 [25] without corticosteroid usage were included. If patients had been on corticosteroids, only those who became non-ambulant at age < 14 years were enrolled. If patients could not understand or answer the questions or write down their answers, we asked the primary caregiver(s) to complete the questionnaire. Respondents were asked to clarify who (patients or representatives) completed the questionnaire.

### Questionnaire survey [Additional file 1]

We distributed a self-reported questionnaire with a linkable anonymized ID to DMD registrants who fulfilled the inclusion criteria, and asked them to complete the questionnaire. The questionnaire was mailed to all patients with DMD who were aged 20 years or older. Responses were collected by mail or electronically using SurveyMonkey between May 2022 and September 2022. Reminders were also mailed to candidates who had not responded by July 2022.

Questionnaire items included: (1) information about developmental disorders (mental retardation, autism Asperger syndrome, attention deficit hyperactivity disorder, and others), (2) school life (regular class or special school, assistant and care, academic achievements, and bullying), (3) information about employment, and (4) information about daily care and physical and financial burdens on the family. Estimated loss of income was based on numbers provided by participants. Questions were either closed-ended or requested concrete numbers. We requested the representatives to only write down the answers if the patients had sufficient ability to answer the questions. If the patients could not answer the questions, then the representatives were asked to answer the questions as the proxy.

### Data analysis

Data are presented as mean ± standard deviation (SD), median, range, frequency, and percentage. All statistical analyses were performed using SPSS for Macintosh (Version 26; SPSS Inc., Chicago, IL).

### Supplementary Information

**Additional file 1.** Questionnaire.

## Data Availability

The data that support the findings of this study are available from the corresponding author (Madoka Mori-Yoshimura) on reasonable request.

## Abbreviations

BMD: Becker muscular dystrophy
COVID-19: Coronavirus disease 2019
DMD: Duchenne muscular dystrophy
NCNP: National Center of Neurology and Psychiatry
NPPV: Noninvasive positive pressure ventilation
Remudy: Registry of Muscular Dystrophy in Japan
SD: Standard deviation
TPPV: Tracheostomy positive pressure ventilation

## Code availability

All codes for data analysis are available from the corresponding author (Madoka Mori-Yoshimura) on reasonable request.
