# Supplementary material for: Social difficulties and care burden of adult Duchenne muscular dystrophy in Japan: a questionnaire survey based on the Japanese Registry of Muscular Dystrophy (Remudy)
Source: Orphanet J Rare Dis. 2024 Apr 30;19:182. doi: 10.1186/s13023-024-03087-z (PMC11061927; doi:10.1186/s13023-024-03087-z)
Supplement: Supplementary file 1 — Additional file 1. Questionnaire. [file 13023_2024_3087_MOESM1_ESM.docx]

| Supplementary Table 1. Requests made to school by parents/patients before or after school entry | | | | |
| --- | --- | --- | --- | --- |
|  |  |  | n | % |
| Requests to school | Granted* | | 148 | 63.2 |
|  |  | Elevator/wheelchair lift (renovation or permission to use if already exists) | 145 | 62.0 |
|  |  | Renovation (e.g., toilet, handrail, slope) | 79 | 33.8 |
|  |  | Consideration of or assistance for transfer | 121 | 51.7 |
|  |  | Other | 25 | 10.7 |
|  | Not granted* |  | 60 | 25.6 |
|  |  | Elevator/wheelchair lift (renovation or permission to use if already exists) | 33 | 14.1 |
|  |  | Renovation (e.g., toilet, handrail, slope) | 18 | 7.7 |
|  |  | Consideration of or assistance for transfer | 7 | 3.0 |
|  |  | Other | 15 | 6.4 |
|  | No request* |  | 54 | 23.1 |
| *multiple answers accepted | | |  |  |

Supplementary material: Questionnaire (originally in Japanese)

Section A. General characteristics

1. How old are you ?
2. Please write down the date of questionnaire completion.
3. If you (the patient) are not able to write down the answers yourself, please specify who your representative will be.
4. How old were you when you started using a wheelchair?
5. How old were you when you lost ambulation?
6. Do you use a ventilator?

Yes/No

If yes, choose from one of the following: part time NPPV/full time NPPV (since age )/TPPV

If yes, how old were you when you started using a ventilator?

Section B. Developmental problems and school life

1. Do you have an intellectual or developmental disorder?

No/Unknown/Yes

If yes, choose from one of the following: intellectual disorders/autism spectrum disorders/learning

disorders/attention deficit, hyperkinetic disorders/other

1. Have you experienced any problematic behavior(s)?

Yes/No

If yes, choose from one of the following: domestic violence/skipping class/suicide attempts/self-mutilation/violent incidents/other

1. What was the last school you graduated from?

Junior high school/high school/university, college/graduate school/currently a university or college student/currently a graduate school student

1. What type of elementary school did you graduate from?

Regular elementary school/elementary school for disabled children due to intellectual reasons/elementary school for disabled children due to physical reasons/elementary school for disabled children due to both intellectual and physical reasons/other

9. If you chose “regular elementary school,” how was your academic performance at the school? Please choose from one of the following:

Good/fair/poor/other

10. Were assistants present (teachers not included) in the elementary school to help you with attendance, eating, using the toilet, and/or transitions on a regular basis?

Yes (dispatched from the Board of Education/mother/father/care worker/other)/No

11. What type of elementary school did you graduate from?

regular elementary school/elementary school for disabled children due to intellectual reasons/elementary school for disabled children due to physical reasons/elementary school for disabled children due to both intellectual and physical reasons/other

12. If you chose “regular elementary school,” how was your academic performance at the school? Please choose one of the following:

good/fair/poor/other

13. Were regular assistants present (teachers not included) in the elementary school to help you with attendance, eating, using the toilet, and/or transitions more than a day?

Yes (dispatched from the Board of Education/mother/father/care worker/other)/No

14. What type of junior high school did you graduate from?

Regular junior high school/junior high school for disabled students due to intellectual reasons/junior high school for disabled students due to physical reasons/junior high school for disabled students due to both intellectual and physical reasons/other

15. Were regular assistants present (teachers are not included) in the junior high school to help you with attendance, eating, using the toilet, and/or transition more than a day?

Yes (dispatched from the Board of Education/mother/father/care worker/other)/No

If yes, how long did they attend to you?

16. What type of high school did you graduate from?

Regular school/high school for disabled students due to intellectual reasons/high school for disabled students due to physical reasons/high school for disabled students due to both intellectual and physical reasons/other

17. Aside from requesting an assistant, did you make individual requests to the school(s) about your handicap due to DMD?

Yes and granted/yes but not granted/no requests

If yes, please specify the request(s) that was granted.

Handrail/elevator or wheelchair lift (construction of new one or permission to use existing one)/other renovation in the school/consideration of or assistance for transfer/other

If yes, please specify the request(s) that was **not** granted.

Handrail/elevator or wheelchair lift (construction of new one or permission to use existing one)/other renovation in the school/consideration of or assistance for transfer/other

18. Have you been bullied? Yes/No

If yes, please specify when you were bullied (multiple answers accepted)

Kindergarten/nursery school

Elementary school

Junior high school

High school

University/professional training college

If yes, please specify whether it happened when you were attending regular classes or not.

If yes, what do you think the reason was for being bullied?

Physical handicap due to muscular dystrophy/communication problems/learning problems/intellectual problems/developmental problems/other

If yes, did the bullying have an influence on your life?

Yes/No

If yes, was the influence positive or negative?

19. Have you ever been employed?

Yes/No

If yes, are you currently employed?

Yes/No

If no, please specify the reason you are not currently employed (physical/not physical)

If no, please specify the reason you have never been employed.

Physical disability due to muscular dystrophy/training/student/intellectual disability/personal reasons/other

20. Have you experienced problematic disorders? Yes/No (skipping class/domestic violence/suicide attempts and/or self-mutilation/violent incidents/drug abuse/other)

21. Have you ever been diagnosed with a developmental disorder?

Yes/No

If yes, choose a disorder from among those listed below.

autistic spectrum disorders/attention deficit hyperkinetic disorders/learning disorders/intellectual disorders/other

22. Do you have any blood-relative cohabitants?

Yes/No

If yes, please choose from among those listed below.*

Father/mother/brother/sister/paternal grandfather/paternal grandmother/maternal grandfather/maternal grandmother/paternal uncle/paternal aunt/maternal uncle/maternal aunt/other

If yes, please specify the primary caregiver.

Father/mother/brother/sister/paternal grandfather/paternal grandmother/maternal grandfather/maternal grandmother/paternal uncle/paternal aunt/maternal uncle/maternal aunt/other

If no, who do you live with?

　　Nobody/spouse/cohabitants who are not relatives/reside in institution or hospital/other

23. Do you use professional care workers?

Yes/No

If yes, what percentage of your care time is handled by professional care workers?

24. How much unpaid care time in 24 hours is provided by family members?

25. Please provide a breakdown of the care time ratio for each family member cohabitant.

26. What is the total household income (including all cohabitants) in Japanese yen? Please choose from the below options.

More than 15 million/more than 10 million and below 15 million/more than 6 million and below 10 million/ more than 4 million and below 6 million/more than 4 million and below 6 million/ more than 2 million and below 4 million/less than 2 million

27. Please provide a breakdown of the family’s household income.

Family income/salary of the patient/welfare/disability pension/other

28. Please estimate the summed annual income of the entire family that could be earned if cohabitants did not spend time on care.
